# Surgical treatment of bilateral vanishing lung syndrome: a case report

**DOI:** 10.1186/s13019-020-01246-4

**Published:** 2020-07-29

**Authors:** Dmitry Borisovich Giller, Galina Vladimirovna Scherbakova, Boris Dmitrievich Giller, Arkadyi Leybovich Khanin, Vladimir Nikolayeich Nikolenko, Mikhail Yegorovich Sinelnikov

**Affiliations:** 1 M.I. Perelman Department of Phthisiopulmonology and Thoracic Surgery, I.M. Sechenov First Moscow State Medical University (Sechenov University), 119991, 8 Trubeckaya str., Moscow, Russia; 2 Department of Phthisiopulmonology, Novokuznetsk State Institute of Postgraduate Medical Education - a branch of FSBEI APE PO RMACPE of the Ministry of Health, 5 Stroiteley Pr, Novokuzneck, 654005 Russia; 3 Human Anatomy Department, I.M. Sechenov First Moscow State Medical University (Sechenov University), 119991, 8 Trubeckaya str, Moscow, Russia; 4 Institute for regenerative medicine, I.M. Sechenov First Moscow State Medical University (Sechenov University), 119991, 8 Trubeckaya str, Moscow, Russia

**Keywords:** Bullous emphysema, Gigantic lung bullae, VATS, Spontaneous pneumothorax, COPD surgery

## Abstract

**Background:**

Volume reduction surgery is a routine treatment method for lung emphysema in chronic obstructive pulmonary disease (COPD) patients. The formation of giant bullous emphysema is an indication for surgical bullectomy. Bilateral giant bullae severely compromise lung function and complicate surgical treatment.

**Case presentation:**

We present the algorithm for surgical treatment and correction of complications in a 38-year-old male with bilateral giant bullae (vanishing lung syndrome), severe COPD. Primarily the patient was admitted with a mild cough, mucopurulent sputum and dyspnea. A CT scan revealed bilateral giant bullae, displacing up to 50% of lung volume. A two-stage surgical bullectomy was planned, yet postoperative complications due to secondary bullae rupture prompted urgent revision with contralateral bullae resection. After complete bullectomy, severely reduced lung volume was successfully managed throughout a long postoperative rehabilitation period. At 5 year follow-up, spirometry indicators and radiological examination show significantly improved and stable lung function.

**Conclusion:**

This clinical case demonstrates the technical difficulties and possible complications of extended bilateral lung resections in patients with severe vanishing lung syndrome. Single-stage treatment of bilateral giant bullous emphysema is recommended to minimize postoperative complications and reduce risk of bullae rupture. Positive long-term outcome outweighs possible complications of surgical treatment.

## Background

Chronic obstructive pulmonary disease (COPD) can present as bullous emphysema, which can lead to the formation of giant bullae. Though this is a rare phenomenon, giant emphysematous bullae, characteristic of “vanishing lung syndrome”, can lead to formation of several giant air pockets, naturally expanding, displacing a significant amount of already compromised lung tissue. This leads to saturation related complications, as well as higher risk of infection and rupturing. Vanishing lung syndrome usually presents as one or several giant emphysematous bullae, and is often accompanied by other lung tissue pathology, including smaller bullae and constrictive pathology. Rupturing of emphysematous type II bullae leads to spontaneous pneumothorax, and is the most frequent indication for emergency thoracic surgery in patients with COPD [1, 2]. Giant bullae should be treated surgically. A large resection bullectomy a spontaneous pneumothorax can be caused by barotrauma due to lung ventilation or insufficient volume of primary lung resection when large bullae were located close to the resection line [3, 4]. This requires thorough preoperative planning to account for possible complications. We present a clinical case of surgical treatment of bilateral giant bullae in a patient with COPD, written according to CARE case report guidelines. The treatment was complicated by a spontaneous pneumothorax due to an undiagnosed bulla underlying the giant emphysematous bulla. Urgent surgical treatment allowed for correction and stabilization of the patient.

## Case presentation

**Fig. 1:**
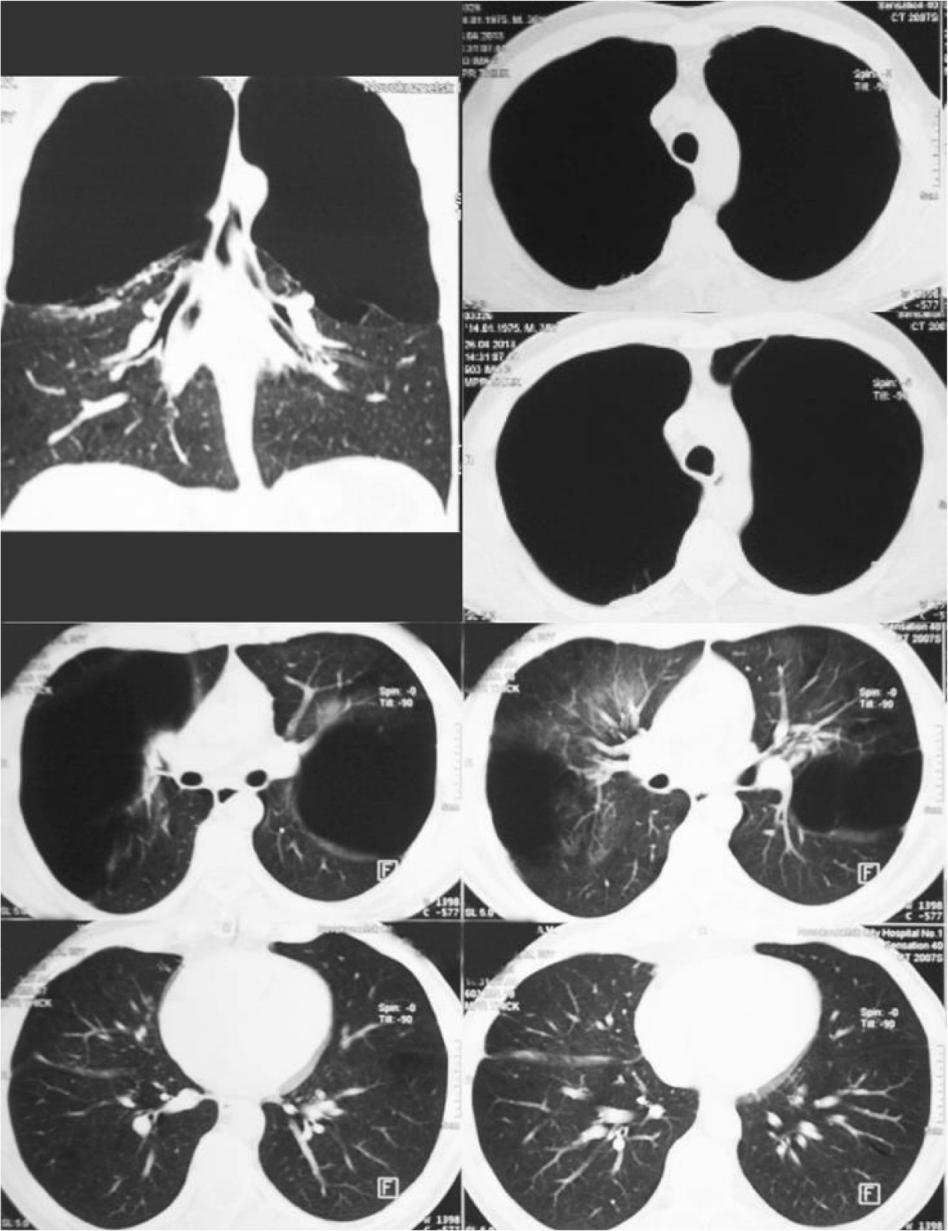
Chest CT scan on admission. Upper sections of the both lungs contains gigantic bullae over ½ of hemithorax volume. Pulmonary parenchyma in middle and lower sections is preserved, but emphysemateus. It contains singular bullae

A 38-year-old male with COPD and bullous pulmonary emphysema was admitted into the phthisiopulmonology department, complaining of a periodic cough with mucopurulent sputum in the morning, whistling in the lungs in horizontal position, shortness of breath during mild exercise, persistent fatigue. The patient had a number of lung health risk factors (such as a work on a mining truck, contact with dust agents of silicates, volatile substances, smoker), with no prior history of lung disease. Physical examination revealed an expiratory wheeze and fine crackles during forced expiration. Radiographically, giant thin-walled bullae were detected in both lungs, characteristic of vanishing lung syndrome [5–7]. Alpha-1 antitripsin deficiency was excluded (α1- antitrypsin levels were normal). Spirometry showed FEV1/VC ratio to be < 0.7. Surgical treatment was advised with prior patient stabilization. Inhalation of Tiotropium bromidе 5 mcg per day was prescribed. At the same time NYHA [8] class III (2 grade MRC) dyspnea persisted. A chest CT showed centrilobular emphysema and bilater giant emphysematous bullae (Fig. 1). Sputum fluorescent microscopy and bacteriological testing did not reveal acid-fast bacilli. Indications for surgical treatment were presented according to ERS/ATS criteria [9], taking into account respiratory failure progression and negative patient dynamics.

**Fig. 2:**
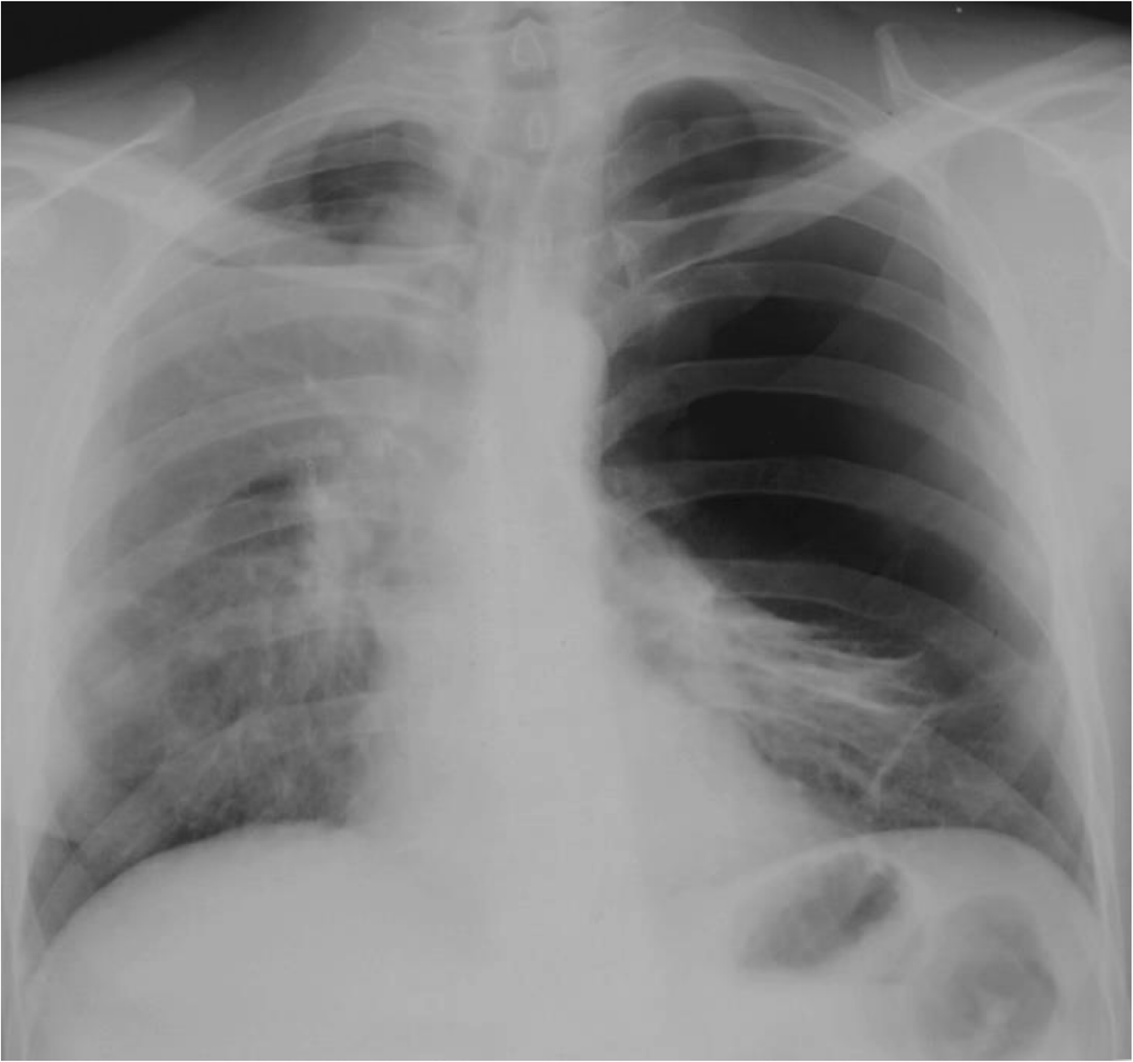
Chest plain film past the first surgery. Giant bull on the right is enlarged and fills about 2/3 of hemithorax volume. Mediastinum is shifted to the right. Right lung is not extended

Surgical protocol consisted of a two-stage approach. Primarily, under combined endotracheal anesthesia with separate bronchial intubation, a VATS right upper lobectomy was performed. Surgery duration was 240 min, blood loss was minimal (30 ml). Complete right upper lobe excision allowed for the removal of a giant bulla (25 cm in diameter). Intraoperatively no complications occurred. 26 h after primary surgery the patient presented with acute respiratory distress, upon examination an acute spontaneous pneumothorax on the right with bullae hyperextension on the left (Fig. 2) was diagnosed. Respiratory failure progression (pCO2 = 37.0 mmHg, pO2 = 58 mmHg) prompted urgent surgery. A revisional surgery on the right and a left lobectomy (S1–2, S3) was planned. During surgery, a type II bulla (7 cm) rupture was revealed in the right lung outside the previous resection line, which caused the pneumothorax, a S6 re-resection was performed with removal of the ruptured bulla. A S1–2, S3 anatomical resection was performed on the left lung with removal of a giant bulla (25 cm in diameter). Surgery duration was 40 min, total blood loss was 40 ml. Postoperatively the patient received respiratory exercise and pharmacological therapy (Olodaterol 5mcg; Tiotropium bromide 5mcg). Due to a large volume of resected lung tissue, slow pulmonary expansion was carried out to reduce the risk of pneumothorax and formation of new bullae. This required lengthy hospitalization with pleural drains removed 30 days after surgery. An artificial pneumoperitoneum aimed on reduction of intrapleural pressure to reduce pneumothorax risk and pulmonary tissue hyperextension.

**Fig. 3:**
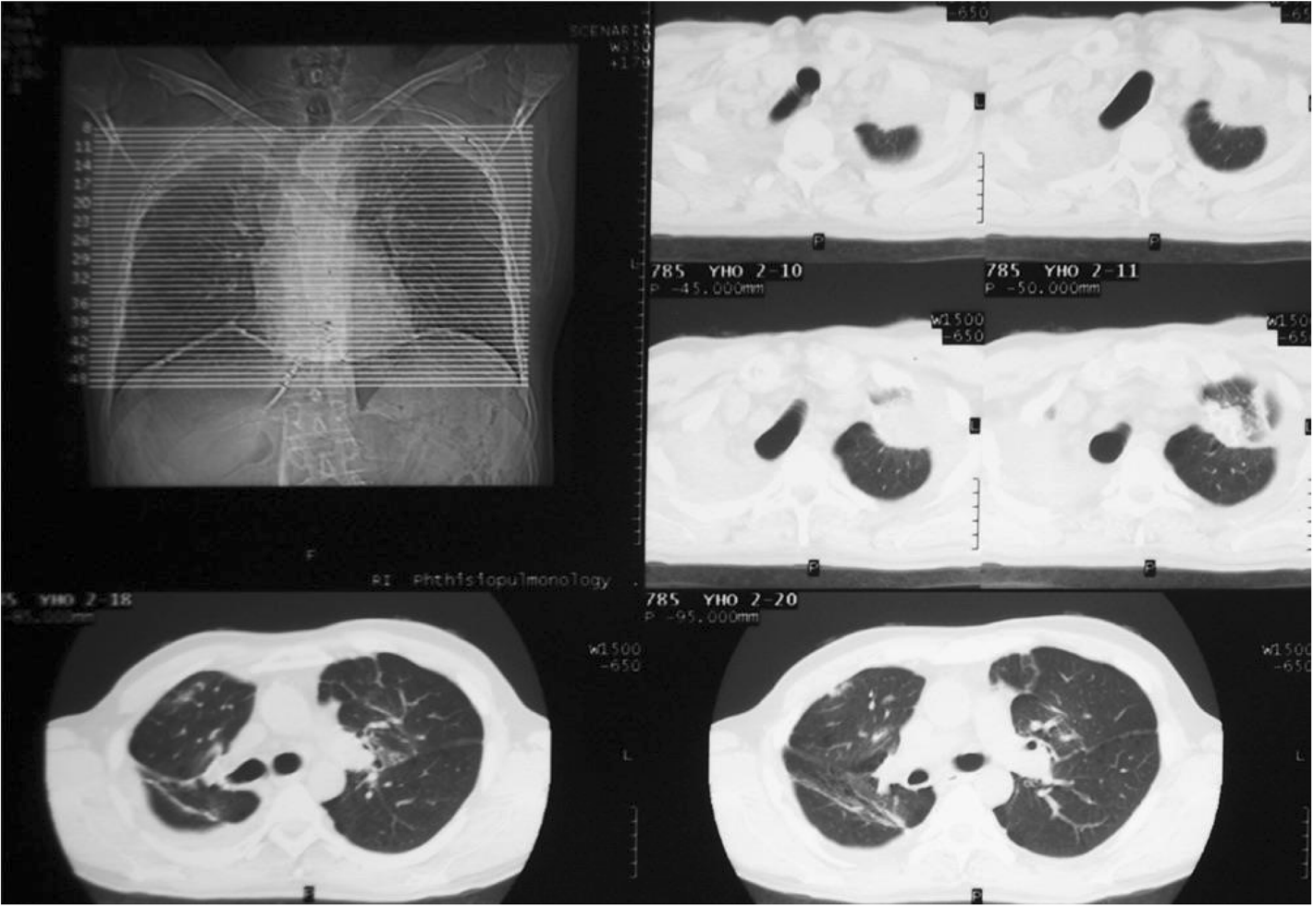
CT scan on discharge. Both operated lungs are extended

**Fig. 4:**
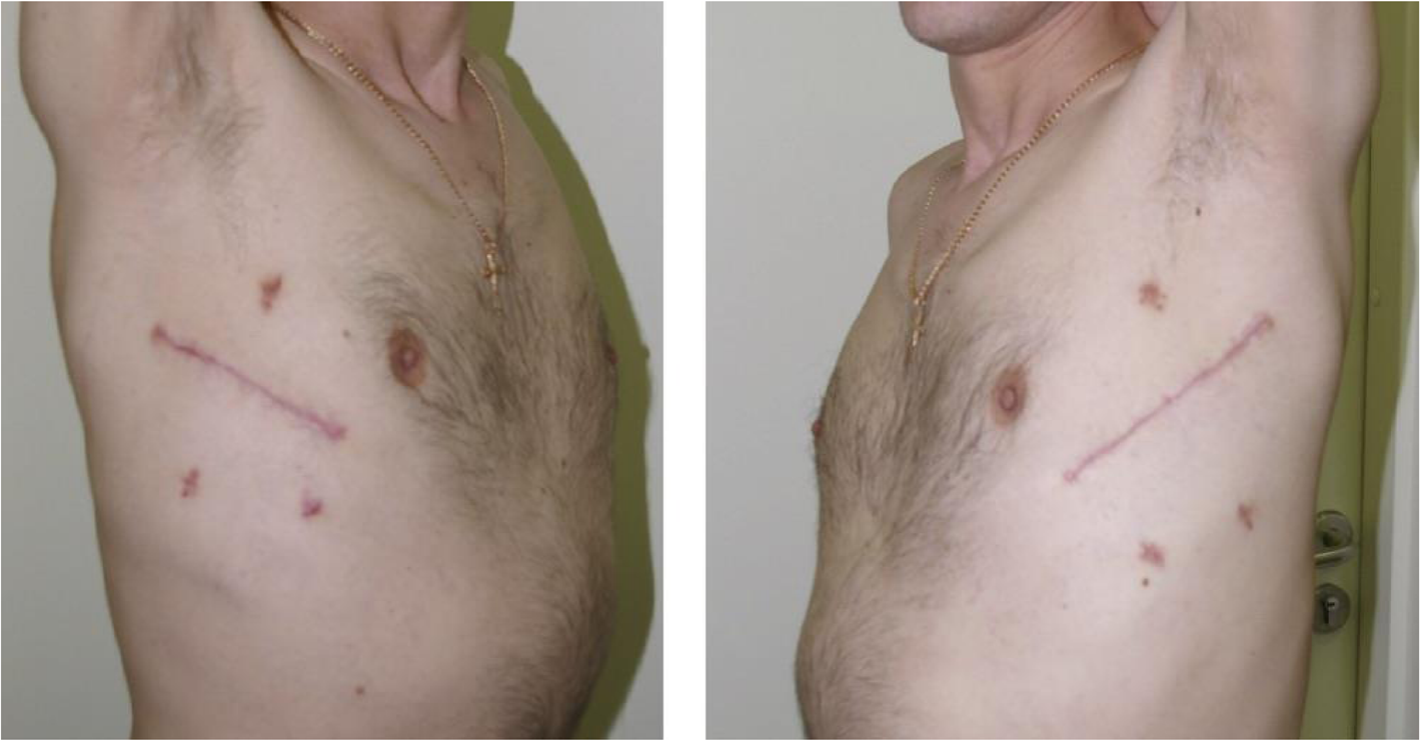
Postoperative scars

The patient was discharged in a satisfactory condition 30 days after surgery, pulmonary pneumatization was satisfactory (Fig. 3). Wound closure was satisfactory (Fig. 4). Bullous emphysema was confirmed histologically. In addition to thin fibrotic bullae, pneumosclerosis and lung congestion was revealed: perivascular lymphoid infiltrates, vascular wall thickening, foci of atelectasis, emphysematous lung tissue, lymphoid tissue inhibition in lymph nodes, sites of fibrosis were identified histologically. Upon discharge, importance of quitting smoking, lung exercise and regular observation were thoroughly discussed with the patient.

**Fig. 5:**
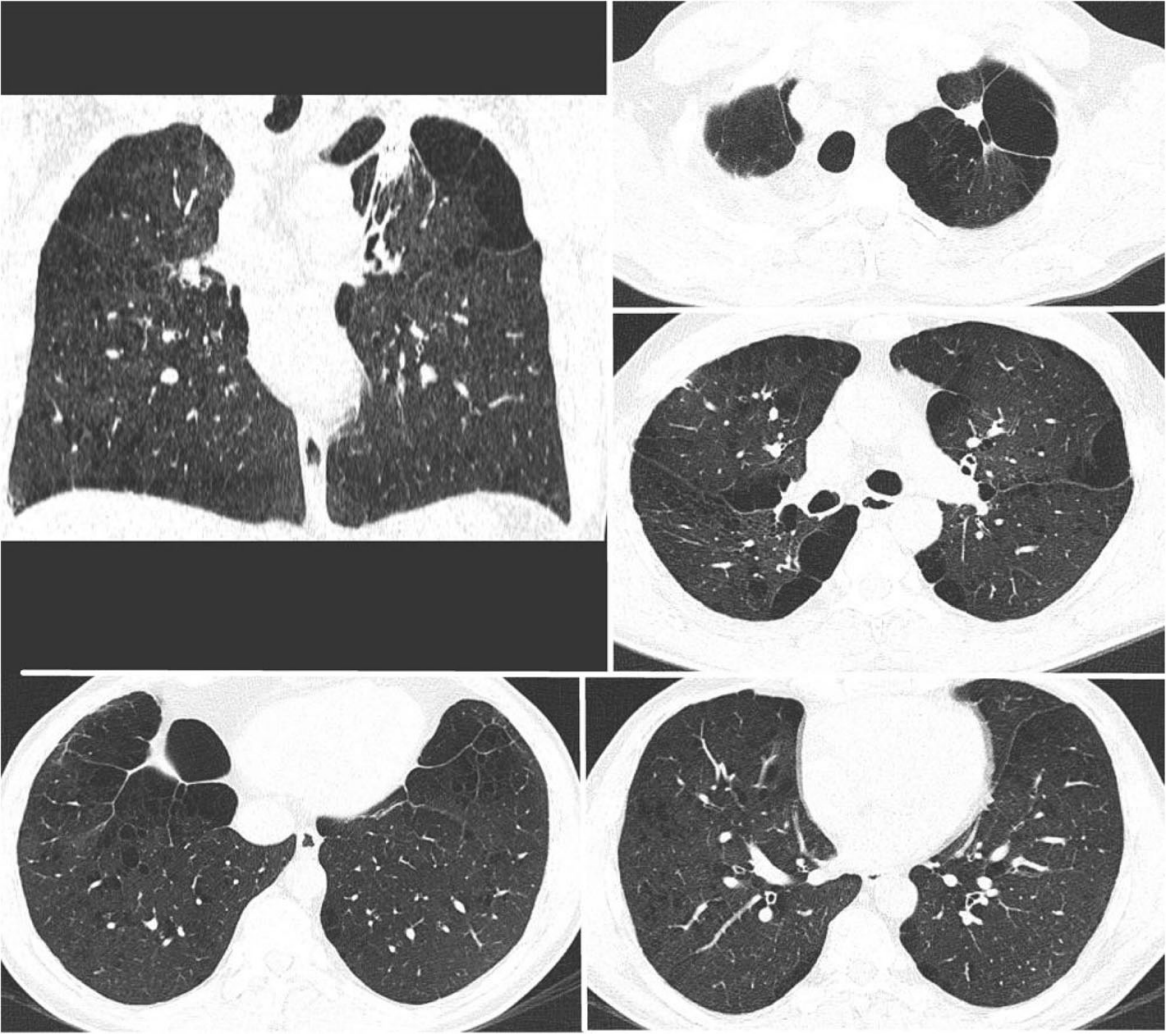
Chest CT scan 6 years past surgery. Both lungs are extended and fulfills hemithorax volume. In upper and middle sections of both lungs bullae up to 6 cm in diameter are visualized

At 5 years follow-up past the patient was smoke-free, physically active, overall condition was above satisfactory. I-II NYHA class dyspnea was present, no II tone accent over the pulmonary artery was present. Fine crackles during forced expiration were revealed mostly on the right. ECG and echocardiogram showed no signs of pulmonary hypertension. Radiological examination showed few small bullae in the lungs, no giant bullae formation was observed (Fig. 5). No additional adverse events except several small bullae were found. Overall improved spirometry results were stable (Table 1).

**Table 1:** Examination results

	Prior surgery	A month past surgery	5 years follow-up
Leukocytes	9,8 × 10^9^/L	15,0 × 10^9^/L	7,9 × 10^9^/L
Arterial-blood gas (ABG)	pCO2 37,4; pO2 73;	pCO2 37,6; pO2 78;	pCO2 36,5; pO2 82;
Spirometry	VC 2, 77 L, 53%; FEV1 1,54 L, 37%; FEV1/VC 69%	VC 1.43 L, 27%;	VC −3.71 L (69%),
	Severe COPD	FEV1 1,51 L, 36%; FEV1/VC 113%	FEV1–1.84 L (45.3%), FEV1/VC = 49,6%, MEF 25/75–1.11 L (25.5%)
		Severe COPD	Severe COPD
6MWT	422 m	403 m	550 m
CAT, mMRC GOLD 2 [10]	mMRC 2	mMRC 1	mMRC 1

## Discussion

The formation of rapidly progressing riant emphysematous bullae is often associated with COPD progression [1, 10–12], and if combined with a FEV1 decrease to less than 25%, may be an indication for lung transplantation [13]. Significant progressive reduction of spirometry indications and reduced exercise tolerance accompanied with dyspnea are characteristic to patients with vanishing lung syndrome (VLS). Surgical treatment is possible and effectively reduces dyspneic symptoms and significantly lowers the risk of spontaneous pneumothorax in patients with VLS [14]. Despite the associated risks of bulla recurrence, VATS bullectomy significantly improves patient respiratory function and reduces risks of serious complications associated with giant emphysematous bullae. Our case reports shows, that within a 5 year follow-up period it is possible that no recurrence of giant bullae manifests, which underlines the efficacy of surgical treatment of patients with VLS.

VLS in patients with COPD is complicated by significant reduction in healthy lung tissue, and is often accompanied by severe respiratory function morbidity. VATS giant bulla resection should be accompanied by proper pharmacological therapy and strict respiratory exercise regimen. Surgical excision of giant bullae significantly reduce the ratio of pleural cavity to lung volume, so we recommend gradual expansion with controlled pneumoperitoneum to reduce risk of postoperative complications and spontaneous pneumothorax.

In the presented case report, primarily a two-stage surgery was planned, but due to postoperative complications, urgent surgery and second stage surgery were performed 26 h after primary surgery. It is important to note, that a single stage bilateral bullectomy is recommended, with simultaneous controlled peritoneum and pleural drainage. This allows to reduce the adverse effects of lung volume reduction.

## Conclusion

In conclusion, surgical resection of giant emphysematous bullae and appropriately prescribed therapy (drug treatment and hygiene regime) allowed us to achieve a positive effect and maintain the patient’s life quality for a long time [15], verified by postoperative respiratory function indicators, and radiological imaging. It is important to plan a single-stage surgical intervention with compensation of lung volume reduction (controlled pneumoperitoneum, pleural cavity draining), appropriate pharmacological therapy and respiratory exercises to maintain the positive effect of VLS treatment.

## Data Availability

Data and material available upon request.

## Abbreviations

COPD: Chronic obstructive pulmonary disease
NYHA: New York Heart Association
CT: Computed Tomography
ERS/ATS: European Respiratory Society/ American Thoracic Society
MRC: Medical Research Council
mMRC: Modified Medical Research Council
VATS: Video Assisted Thoracic Surgery
ECG: Electrocardiography
6MWT: Six Minutes Walking Test
GOLD: Global Inititiative For Chronic Obstructive Lung Disease
